# Association between Circulating Ectodysplasin A and Diabetic Kidney Disease

**DOI:** 10.1155/2023/5087761

**Published:** 2023-04-12

**Authors:** Xia Deng, Chang Guo, Huijuan Qin, Li Zhao, Yanyan Li, Zhicong Zhao, Haoxiang Li, Ling Yang, Dong Wang, Guoyue Yuan

**Affiliations:** ^1^Department of Endocrinology, Affiliated Hospital of Jiangsu University, Zhenjiang, Jiangsu, China; ^2^Department of Nephrology, Affiliated Hospital of Jiangsu University, Zhenjiang, Jiangsu, China

## Abstract

**Background:**

Ectodysplasin A (EDA), a member of the TNF family, plays important roles in ectodermal development, while recent studies expanded its regulatory effects on insulin resistance and lipid metabolism. This study was the first time to investigate the correlation between circulating EDA and albuminuria in patients with T2DM.

**Methods:**

A total of 189 T2DM and 59 healthy subjects were enrolled in the study. We analyzed the concentrations of EDA by ELISA. Plasma glucose, insulin, HbA1c, lipids, creatinine, BUN, and UACR were also measured. Insulin resistance and pancreatic cell function were assessed by HOMA.

**Results:**

Circulating EDA concentration was significantly increased in T2DM patients and increased with the degree of albuminuria. EDA was positively correlated with age, FIns, HOMA-IR, HOMA-*β*, Scr, and UACR, and negatively correlated with eGFR. Linear stepwise regression showed that FIns, HOMA-*β*, and UACR were independent influencing factors of EDA. Logistic regression analysis showed that EDA was independently associated with the occurrence of albuminuria in T2DM. ROC curve showed that EDA had an area under the receiver operating curve of 0.701 [95%CI = (0.625 − 0.777), *P* < 0.001].

**Conclusion:**

EDA is positively correlated with the degree of albuminuria in patients with T2DM and may be involved in the occurrence and progression of diabetic kidney disease (DKD).

## 1. Introduction

Diabetic kidney disease (DKD) is a common complication of diabetes, and it is also the main cause of the end-stage renal disease (ESRD) [1]. Long-term uncontrolled diabetes exposes a variety of renal cells including endothelial cells (ECs), smooth muscle cells (SMCs), mesangial cells (MCs), and podocytes to high glucose toxicity, leading to increased advanced glycation products (AGEs), activation of protein kinase C (PKC), increased expression of transforming growth factor (TGF-*β*), and reactive oxygen species (ROS) production [2]. At the same time, the abnormal activation of the renal local renin-angiotensin system (ROS) also changes hemodynamics. The combined action of these factors leads to excessive proliferation and hypertrophy of MCs, accumulation of extracellular matrix (ECM) proteins, thickening of glomerular basement membrane (GBM), and finally, renal pathological changes such as interstitial fibrosis and glomerulosclerosis occurs [3]. DKD is widely characterized by persistent high albuminuria and a subsequent decline in the glomerular filtration rate (GFR). With the irreversible progressive injury of renal function, it finally enters the stage of uremia [4]. Renal pathology is the most accurate method for the diagnosis of DKD, but it is not easy to obtain. Vicariously, urinary albumin creatinine ratio (UACR) is often used in the clinical diagnosis and staging of DKD [5].

Ectodysplasin A (EDA) belongs to the tumor necrosis factor (TNF) family [6], and its gene is located in the long arm of the X chromosome [7]. EDA transcripts can form multiple EDA subtypes after complex shearing, but only two subtypes (EDA1 and EDA2) have been found to bind to their respective receptors and have biological activities [8, 9]. In previous studies, EDA was considered to be involved in ectodermal tissue development by stimulating effectors or inhibitors of pathways like Wnt, fibroblast growth factor (FGF), and TGF-*β* mediated by NF-*κ*B [6]. Classically, EDA gene defects are thought to be associated with the occurrence of X-linked hypohidrotic ectodermal dysplasia (XLHED) [7]. In recent studies, researchers have newly discovered the biological role of EDA in metabolic diseases; that is, EDA may be involved in the regulation of muscle insulin sensitivity and liver lipid metabolism and considered it as a novel hepatokine [10–12]. The previous study found that the serum EDA level in newly diagnosed type 2 diabetic patients increased significantly [13]. These new findings have not only aroused the discussion of some researchers but also triggered some new thinking. Considering the relationship between EDA and FGF, TGF-*β* and other cytokines, and its involvement in the regulation of glucose and lipid metabolism, we speculated that EDA might be related to DKD. Therefore, we conducted this study to explore the correlation between circulating EDA concentration and albuminuria in T2DM patients, and to evaluate the diagnostic value of EDA in T2DM patients with albuminuria.

## 2. Materials and Methods

### 2.1. Subjects

In this cross-sectional study, we recruited 189 T2DM patients and 59 healthy controls. The definition of T2DM is based on the 1999 WHO diagnostic criteria [14]. It is worth noting that the subjects have the following situations: (1) type 1 diabetes; (2) acute diabetic complications; (3) chronic viral or bacterial infection; (4) other severe kidney diseases and drug-induced kidney diseases; (5) severe liver disease; (6) autoimmune diseases; (7) tumor; (8) psychosis; and (9) pregnancy were excluded. DKD was defined as UACR ≥ 30 mg/g and/or eGFR < 60 mL/min/1.73 m^2^ in the absence of signs or symptoms of other primary causes of kidney damage. In addition, in order to reduce the effect of renal failure on EDA, all T2DM patients with eGFR less than or equal to 30 mL/(min^∗^1.73m^2^) were excluded from this study. Data on the use of hypoglycemic drugs, lipid-lowering drugs, angiotensin-converting enzyme inhibitors (ACEI), and/or angiotensin receptor blockers (ARBs) in the past three months were obtained from the clinical records of each patient. Microalbuminuria and macroalbuminuria are usually determined by clinical indicators (UACR). T2DM patients with UACR ≤ 30 mg/g were defined as normal albuminuria group (*n* = 97), T2DM patients with UACR of 30-300 mg/g were defined as microalbuminuria group (*n* = 62), and T2DM patients with UACR ≥ 300 mg/g were defined as macroalbuminuria group (*n* = 30). Renal function was assessed by eGFR using the simplified kidney disease equation (sMDRD) diet correction method, as follows: eGFR = 186.3 × (serum creatinine)^−1.154^ × (age)^−0.203^ × [(0.742) if female] [15]. The study was approved by the biomedical research ethics committee of the Affiliated Hospital of Jiangsu University in Zhenjiang, China, and was carried out in accordance with the Declaration of Helsinki. All participants informed consent to the purpose of this study.

### 2.2. Collection of Clinical and Biochemical Data

The general clinical data of patients were collected, including age, gender, height, weight, blood pressure, waist circumference (WC), and hip circumference (HC). WHR is calculated as the ratio of WC to HC, and BMI is expressed as weight per square kilometer (kg/m^2^). Blood urea nitrogen (BUN) was measured by rate method, serum creatinine (Scr) was measured by enzyme method, and UACR was measured by immunoturbidimetry. The glucose oxidase method was used to determine the blood glucose level, and the chemiluminescence method was used to determine the levels of insulin and C-peptide. Glycosylated hemoglobin (HbA1c) was determined by high-performance liquid chromatography (HPLC) (ARKRAY company, Kyoto, Japan). The enzymatic method is used to determine blood lipid profile, including high-density lipoprotein cholesterol (HDL-C), low-density lipoprotein cholesterol (LDL-C), total cholesterol (TC), and triglyceride (TG) parameters (Beckman Coulter Inc.). Insulin resistance was assessed by homeostasis model assessment (HOMA): HOMA − IR = fasting plasma insulin (FIns) × fasting blood glucose (FPG)/22.5; HOMA − *β* = 20 × FIns (*μ*U/mL)/FPG (mmol/L) − 3.5) (%).

### 2.3. Estimation of Serum EDA Concentration

The collected blood samples were centrifuged (1500 g, 4°C, 20 min); then, each serum sample was separated and labeled and immediately stored in a refrigerator at -80°C. Serum EDA concentrations were measured by a commercial enzyme-linked immunosorbent assay kit (ELISA) (EIAab Science Inc., Wuhan, China; Catalog number E1976h). Detection range: 78-5000 pg/mL, minimum detection limit: 20 pg/mL. The intraassay CV was ≤7.8%, and the interassay CV was ≤8.9%.

### 2.4. Statistical Analysis

All statistical analyses were performed using SPSS version 20.0. For normally distributed data, continuous variables are expressed as mean ± standard deviation (M ± SD); for skew distribution data, continuous variables are expressed as median [quartile (IQR)]; for categorical variables, the data are expressed as frequency (*n*) and percentage (%). One-way ANOVA was used to compare data between groups. In the unadjusted model, the correlation coefficients of EDA and other clinical indicators were evaluated by the Pearson or the Spearman correlation analysis. The independent influencing factors of EDA were determined by multiple stepwise linear regression analysis. Linear regression analysis was used to evaluate the influencing factors of UACR. Multiple binary logistic regression model was used to analyze the correlation between EDA and albuminuria (UACR ≥ 30 mg/g), and ROC curve was used to evaluate the predictive value of EDA for albuminuria.

## 3. Results

### 3.1. Circulating EDA Increased in T2DM and Was Related to the Degree of Albuminuria

We first described the clinical characteristics of all subjects (Table 1). No difference in sex ratio was found between the groups. Compared with the control group, T2DM patients have higher prevalence of hypertension (manifested in the increase of SBP and DBP) and greater age; the level of BMI, WHR, FPG, 2hPG, FINs, HbA1C, HOMA-IR, TG, ALT, BUN, eGFR, and EDA was significantly increased; and the level of 2hCP, HOMA-*β*, HDL-C, and creatinine was significantly reduced (*P* < 0.05). There was no statistically significant difference in other indicators.

**Figure 1:**
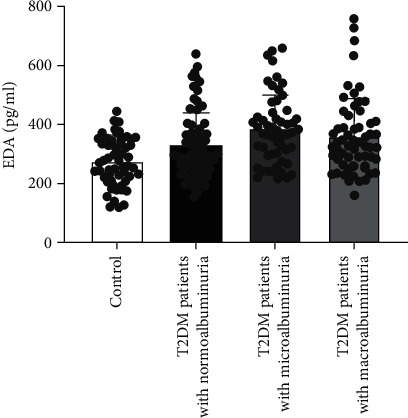
Circulating EDA concentration between different subgroups.

Further analysis of data with significant differences in Table 2, we described the baseline clinical characteristics among all subgroups of T2DM. With the increase of microalbuminuria, the subjects had a longer course of diabetes, a higher proportion of hypertension history, a higher rate of insulin use, and the level of FIns, SBP, HOMA-*β*, BUN, and creatinine was gradually increased, while eGFR gradually decreased. It is worth noting that the concentration of EDA gradually increased among the normoalbuminuria group, microalbuminuria group, and macroalbuminuria group, and the concentration difference between the groups was statistically significant (Figure 1).

### 3.2. The Correlation between EDA and Clinical and Biochemical Parameters

**Figure 2:**
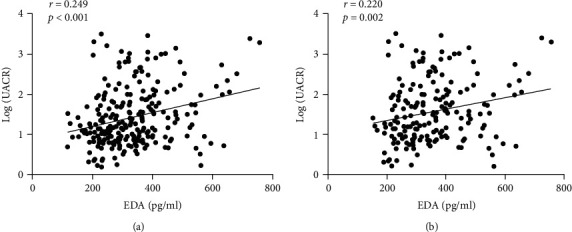
Correlation between serum EDA levels and UACR levels in subjects. (a). Correlation between serum EDA levels and UACR levels in all subjects (*r* = 0.249, *P* < 0.001). (b). Correlation between serum EDA level and UACR level in T2DM patients (*r* = 0.220, *P* = 0.002). The logarithm-transformed values of UACR are used for analysis. (*r* = 0.703, *P* < 0.001).

Next, we studied the correlation between circulating EDA concentration and clinical biochemical parameters (Table 3 and Figure 2). In all subjects, circulating EDA concentration was positively correlated with age, FIns, HOMA-*β* and UACR, but negatively correlated with TG. In T2DM subjects, circulating EDA concentration was positively correlated with age, FIns, HOMA-IR, HOMA-*β*, Scr, and UACR, but negatively correlated with eGFR. In the control group, no statistically significant correlation between circulating EDA and clinical biochemical parameters was observed. Stepwise multiple linear regression analysis showed that FIns, HOMA-IR, and UACR had significant effects on circulating EDA concentration (Table 4).

### 3.3. Results of Multiple Linear Regression Analysis for UACR in T2DM Patients

After that, we took age, duration of T2DM, BMI, and other indicators as independent variables and UACR as dependent variables to conduct multiple linear regression analysis. The results showed that the levels of EDA, SBP, FPG, TC, and creatinine were significantly independent effects of UACR. When the regression equation was further adjusted by sex, smoking, hypertension, and drug use, it was found that the above same variables were still significantly correlated with the changes in UACR (Table 5). These findings suggested that UACR elevated with the increase of EDA, SBP, FPG, and creatinine.

### 3.4. EDA and the Risk of Albuminuria in T2DM

We grouped the circulating EDA concentration of T2DM subjects according to the tertile (*n* = 63 in each group) and studied the effect of different levels of circulating EDA concentration on albuminuria in T2DM patients (Table 6). Firstly, in model 1 (unadjusted), the middle and upper tertile groups were more prone to albuminuria than the lower tertile group [OR = 2.219, 95%CI = (1.075 − 4.582); OR = 3.739, 95%CI = (1.788 − 7.820), respectively], and the higher the concentration of EDA, the greater the risk of albuminuria. Then, after adjusting for age, gender, course of the disease, SBP, and DBP (model 2), we still found the same trend [OR = 2.477, 95%CI = (1.060 − 5.791); OR = 4.112, 95%CI = (1.736 − 9.739), respectively]. After adjusting for multiple factors (model 3), the risk of albuminuria in the middle and high upper groups was still significantly increased [OR = 3.327, 95%CI = (1.302 − 8.499); OR = 5.315, 95%CI = (2.000 − 14.129), respectively].

### 3.5. Predictive Value of EDA for Albuminuria inT2DM

**Figure 3:**
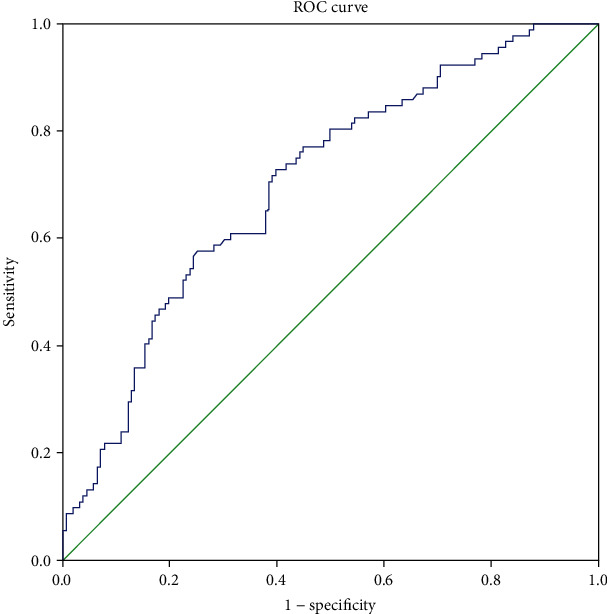
Receiver operating characteristic (ROC) curves for the ability of EDA to discriminate the presence of albuminuria. The ability of the ROC curve based on EDA levels to predict the presence of albuminuria was 0.701 (0.625-0.777). Optimal cutoff points for EDA were 310.47 pg/mL and sensitivity and specificity were 72.8% and 60.3%, respectively.

Finally, we analyzed the value of circulating EDA concentration in the diagnosis of albuminuria in T2DM. ROC curve showed that AUC was 0.701 [95%CI = (0.625 − 0.777), *P* < 0.001], indicating that circulating EDA concentration had diagnostic value for albuminuria in T2DM. Optimal cutoff points for EDA were 310.47 pg/mL and sensitivity and specificity were 72.8% and 60.3%, respectively (Figure 3).

## 4. Discussion

In this study, we found that the circulating EDA concentration in T2DM patients was significantly higher than that in the control group. Consistent with this, there was a positive correlation between circulating EDA concentration and age, FIns, HOMA-*β*, and UACR. Further, we found that the circulating EDA concentration increased with the degree of albuminuria in T2DM patients. At the same time, circulating EDA concentration was also positively correlated with FIns, HOMA-IR, HOMA-*β*, Scr, and UACR, but negatively correlated with eGFR. These results suggest that EDA may be associated with the occurrence and progression of DKD. The tertile grouping showed that EDA could significantly increase the risk of albuminuria in T2DM patients. The ROC curve shows that EDA has a high diagnostic value for albuminuria in T2DM patients. So far as we know, this is the first discovery of the association between EDA and DKD.

In previous studies, EDA has been widely concerned in the research of XLHED and other diseases because of its participation in ectodermal tissue development [7]. However, several recent studies have revealed its importance in metabolic diseases. Awazawa et al. [10] showed that overexpression of EDA resulted in higher JNK phosphorylation level in skeletal muscle, accompanied by the corresponding upregulation of Ser307 phosphorylation level of IRS1, which means the decrease of insulin sensitivity in skeletal muscle. Consistent with this, overexpression EDA mice showed a trend of decreased energy consumption and showed higher plasma glucose levels in glucose tolerance experiments. Correspondingly, knockout of EDA increased skeletal muscle glucose uptake in db/db mice in the hyperinsulinemic-euglycemic clamp experiment and decreased plasma glucose in the insulin tolerance experiment, indicating that EDA knockout can improve skeletal muscle insulin sensitivity. It should be noted that the study of Bayliss et al. [12] got inconsistent results. They found that there was no significant correlation between liver and plasma EDA and insulin resistance markers (such as HOMA-IR, FPG, and FIns) or type 2 diabetes. The degree of obesity in the subject population seems to be an explanation. The results of our study are partly consistent with those of Awazawa et al. We found that the circulating EDA concentration in T2DM patients was positively correlated with fasting/postprandial insulin and HOMA-IR, suggesting that the higher the circulating EDA concentration in T2DM patients, the more serious the insulin resistance. DKD is characterized by changes in GFR and albuminuria. The metabolic abnormalities caused by long-term hyperglycemia and hemodynamic changes due to excessive activation of the local RAS system are important pathogenesis of DKD [2]. The correlation between EDA and DKD may be related to lowering insulin sensitivity and leading to higher plasma glucose.

In addition to hyperglycemia and insulin resistance, hypertension, dyslipidemia, smoking, drinking history, and other factors will lead to progressive renal dysfunction and microcirculatory disorder, which will aggravate the occurrence and development of diabetic nephropathy [16]. Cui et al. found that the circulating EDA level of NAFLD patients was significantly higher than that of healthy subjects [11], which was further confirmed by Bayliss et al. [12]. With the increase in the degree of liver lesions, the concentration of circulating EDA gradually increased and was negatively correlated with HDL-C [11]. Although renal insufficiency may lead to atherosclerotic dyslipidemia due to the loss of certain lipoproteins along with urine, abnormal lipid accumulation in the kidney also plays an important role in the occurrence and progression of various chronic renal insufficiency, especially DKD [17]. Therefore, there is an interaction between abnormal lipid metabolism and DKD. HDL-C can transport cholesterol from extrahepatic tissues to the liver for metabolism and is an important antiatherosclerotic lipoprotein [18]. A 9-year clinical follow-up study showed that low baseline level and subgroup changes of HDL-C could predict the occurrence of DKD [19]. In this study, we did not find a correlation between circulating EDA concentration and HDL-C. But, in all subjects, we figured out that the circulating EDA concentration was negatively correlated with the TG level, which was similar to the research results of Cui et al. [11], possibly due to the use of statins, the significance disappeared in T2DM subgroups. EDA knockout can significantly reduce the accumulation of lipids in HepG2 cells, accompanied by the increase of key fatty acid oxidase gene expression. Similarly, EDA knockout significantly reduced liver lipid droplets in HFD mice [11]. Therefore, EDA plays an important role in regulating lipid metabolism. Although the correlation between EDA and other circulating lipid components may need further exploration, in general, whether EDA is involved in the occurrence of macroangiopathy and microangiopathy by regulating lipid components is a worthy research direction. Consistent with Cui et al. [11], we also observed that circulating EDA concentration was positively correlated with age.

The Wnt/*β*-catenin signaling pathway is involved in regulating interactions of cells, including cell polarization and basement membrane synthesis, thus mediating a variety of physiological and pathological processes, such as inflammation, angiogenesis, and fibrosis [20]. Previous studies have shown that the EDA-EDAR signal is an important factor affecting the typical Wnt signal in the development of skin appendages. By stimulating the transcription of effectors or inhibitors of NF-*κ*B-mediated Wnt, FGF, and TGF-*β* pathways, it regulates the interactions within or between epithelial cells and mesenchymal cells [6, 21]. Research showed that the Wnt/*β*-catenin signaling pathway plays a key role in diabetic kidney injury, especially MCs, podocytes, and tubular cell injury and participates in renal interstitial fibrosis and glomerulosclerosis [22, 23]. A variety of FGF factors are considered to be associated with diabetic renal interstitial fibrosis and albuminuria [24, 25], and TGF-*β* is also an important regulator of excessive accumulation of ECM protein in DKD [26, 27]. Furthermore, EDA2R was also found to be highly expressed in podocytes treated with high glucose concentration in vitro and in glomerular podocytes of diabetes patients. EDA2R may aggravate the development of diabetic nephropathy by regulating podocyte apoptosis and dedifferentiation and increasing ROS production [28]. Therefore, circulating EDA may promote the progression of renal injury in diabetes by acting on EDA2R receptors and regulating these signaling pathways. We will pay attention to this in the follow-up research.

Admittedly, there are some deficiencies in this study, such as the effects of hypoglycemic and lipid-lowering drugs on some biochemical parameters. In the follow-up study, we will include more newly diagnosed T2DM populations and conduct corresponding basic experiments to explore the specific mechanisms. In summary, this study first revealed the correlation between EDA and DKD. EDA is associated with insulin resistance and dyslipidemia in T2DM patients and has good predictive value for albuminuria in T2DM patients. These results suggest that EDA may be involved in the occurrence and progression of DKD.

## Figures and Tables

**Table 1:** Baseline clinical characteristics of all patients.

Characteristics	Control (*n* = 59)	T2DM (*n* = 189)	*P* value
Sex, men (%)	27 (45.8%)	104 (55.0%)	0.214
Age, y	48.02 ± 11.90	55.24 ± 12.46	<0.001
BMI (kg/m^2^)	23.20 (21.30, 25.39)	25.17 (22.93, 27.35)	<0.001
WC (cm)	84.01 ± 8.94	90.74 ± 8.87	<0.001
HC (cm)	96.00 (92.20, 100.50)	96.00 (92.00, 100.50)	0.805
WHR	0.87 (0.82, 0.92)	0.94 (0.90, 0.97)	<0.001
Duration of T2DM (M)	—	48.00 (1.00, 126.00)	—
Hypertension, *n* (%)	4 (6.8%)	90 (47.6%)	<0.001
SBP (mmHg)	118.00 (106.00, 126.00)	132 (122.00, 146.00)	<0.001
DBP (mmHg)	68.00 (61.00, 78.00)	81.00 (75.00, 89.00)	<0.001
Smoking, *n* (%)	15 (25.4%)	45 (23.8%)	0.801
ACEI/ARB use	—	59 (31.2%)	—
Statins use	—	4 (2.1%)	—
Insulin	—	57 (30.2%)	—
Oral agents	—	65 (34.4%)	—
Insulin+oral agents	—	31 (16.4%)	—
FPG (mmol/L)	4.60 (4.26, 5.04)	9.95 (7.94, 12.76)	<0.001
2hPG (mmol/L)	6.36 (5.77, 6.91)	19.05 (15.91, 22.90)	<0.001
FIns (*μ*IU/mL)	4.62 (2.73, 6.56)	7.41 (4.60, 11.84)	<0.001
2hIns (*μ*IU/mL)	26.99 (18.58, 43.56)	28.30 (16.47, 52.38)	0.577
FC-P (ng/mL)	2.17 ± 0.80	2.42 ± 1.15	0.058
2hCP(ng/mL)	7.78 (5.75, 9.64)	4.62 (3.11, 6.78)	<0.001
Hb1Ac (%)	5.70 (5.60, 5.90)	9.30 (8.05, 11.00)	<0.001
HOMA-IR	0.95 (0.56, 1.35)	3.53 (2.17, 4.93)	<0.001
HOMA-*β*	77.02 (46.49, 135.73)	22.70 (11.82, 45.22)	<0.001
TG (mmol/L)	1.30 (0.90, 1.77)	1.93 (1.36, 2.98)	<0.001
TC (mmol/L)	4.92 (4.50, 5.51)	4.94 (4.21, 5.91)	0.673
LDL-C (mmol/L)	2.66 (2.34, 3.30)	2.81 (2.33, 3.65)	0.229
HDL-C (mmol/L)	1.41 (1.19, 1.69)	1.07 (0.90, 1.27)	<0.001
ALT (U/L)	15.40 (9.80, 28.90)	22.00 (13.55, 38.05)	0.014
AST (U/L)	17.10 (13.70, 21.60)	17.40 (12.95, 26.50)	0.998
UA (*μ*mol/L)	285.54 ± 79.74	298.13 ± 87.31	0.325
BUN (mmol/L)	4.70 (4.14, 5.83)	5.19 (4.33, 6.08)	0.045
Creatinine (*μ*mol/L)	66 (58.80, 75.20)	57.80 (47.95, 69.45)	0.001
eGFR(mL/[min1.73m^2^])	103.20 (92.50, 111.60)	110.57 (96.15, 126.20)	0.030
EDA	268.72 (218.31, 331.61)	337.47263.89, 407.00)	<0.001

**Table 2:** Baseline clinical characteristics between all subgroups of T2DM.

Characteristics	T2DM with normoalbuminuria (*n* = 97)	T2DM with microalbuminuria (*n* = 62)	T2DM with macroalbuminuria (*n* = 30)	*P* value
Sex, men (%)	54 (55.7%)	32 (51.6%)^∗^	18 (60%)^∗^	0.739
Age, y	51.07 ± 11.94	59.35 ± 11.86^∗^	60.20 ± 10.89^∗^	<0.001
BMI (kg/m2)	24.77 (22.76, 27.41)	25.34 (23.41, 27.41)	24.65 (21.81, 27.34)	0.462
WC (cm)	90.01 ± 9.62	91.61 ± 7.76	91.30 ± 8.53	0.502
HC (cm)	96.00 (90.50, 100.00)	97.25 (94.00, 102.00)	95.00 (90.75, 105.50)	0.164
WHR	0.94 (0.90, 0.97)	0.94 (0.92, 0.97)	0.95 (0.89, 0.97)	0.965
Duration of T2DM (M)	6.00 (0.50, 81.00)	114.00 (24.00, 183.00)^∗^	120.00 (54.00, 222.00)^∗^	<0.001
Hypertension, *n* (%)	29 (29.9%)	41 (66.1%)^∗^	20 (66.7%)^∗^	<0.001
SBP (mmHg)	127.00 (119.00, 138.00)	140.00 (130.00, 150.00)^∗^	147.00 (126.00, 158.00)^∗^	<0.001
DBP (mmHg)	80.00 (76.00, 87.00)	82.00 (76.00, 90.00)	83.00 (72.00, 92.00)	0.617
Smoking, *n* (%)	24 (24.7%)	15 (24.2%)	6 (20%)	0.865
ACEI/ARB use	22 (22.7%)	26 (41.9%)^∗^	11 (36.7%)	0.030
Statins use	1 (1.03%)	3 (4.8%)	0 (0%)	0.045
Insulin	15 (15.5%)	25 (40.3%)^∗^	17 (56.7%)^∗^	<0.001
Oral agents	18 (18.6%)	36 (58.1%)^∗^	11 (36.7%)	<0.001
Insulin+oral agents	13 (13.4%)	14 (22.6%)^∗^	4 (13.3%)	0.001
FPG (mmol/L)	10.50 (8.17, 13.27)	9.68 (7.59, 12.31)	8.75 (7.23, 12.75)	0.167
2hPG (mmol/L)	19.74 (16.60, 24.00)	19.21 (16.18, 22.05)	16.52 (11.88, 20.19)^∗^	0.043
FIns (*μ*IU/mL)	6.45 (4.37, 10.10)	8.99 (5.49, 15.02)^∗^	9.88 (4.13, 13.03)	0.041
2hIns (*μ*IU/mL)	26.60 (14.47, 45.80)	33.41 (17.00, 53.63)	41.18 (21.96, 55.82)	0.057
FC-P (ng/mL)	2.61 ± 1.10	2.20 ± 1.24	2.30 ± 1.08	0.072
2hCP (ng/mL)	5.41 (3.73, 7.36)	3.85 (2.68, 5.99)^∗^	4.46 (3.00, 5.36)^∗^	0.002
Hb1Ac (%)	9.20 (8.00, 10.90)	9.70 (8.28, 11.25)	8.55 (7.18, 10.65)	0.148
HOMA-IR	3.35 (2.10, 4.32)	3.89 (2.48, 5.44)	3.91 (1.63, 5.46)	0.083
HOMA-*β*	17.34 (10.53, 37.81)	25.30 (15.79, 58.82)	30.14 (14.58, 57.34)^∗^	0.036
TG (mmol/L)	2.05 (1.33, 2.98)	1.70 (1.43, 2.58)	2.00 (1.29, 3.88)	0.620
TC (mmol/L)	4.86 (4.21, 5.67)	4.98 (4.13, 5.90)	5.05 (4.37, 6.26)	0.296
LDL-C (mmol/L)	2.79 (2.35, 3.47)	2.92 (2.31, 3.68)	2.89 (2.32, 3.83)	0.833
HDL-C (mmol/L)	1.03 (0.86, 1.23)	1.12 (0.92, 1.28)	1.16 (0.91, 1.38)	0.192
ALT (U/L)	23.50 (16.55, 40.80)	20.55 (11.90, 34.75)	17.80 (9.53, 26.28)^∗^	0.021
AST (U/L)	18.80 (13.45, 29.15)	15.80 (13.00, 25.40)	14.85 (10.98, 22.33)	0.151
UA (*μ*mol/L)	288.60 ± 80.46	303.47 ± 99.88	317.93 ± 78.92	0.232
BUN (mmol/L)	4.99 (4.27, 5.70)	5.31 (4.33, 6.26)	6.53 (4.80, 8.63)^∗^	0.003
Creatinine (*μ*mol/L)	56.40 (46.85, 65.10)	58.00 (46.38, 70.68)^∗^	73.05 (57.30, 105.98)^∗^^†^	<0.001
eGFR (mL/[min1.73m^2^])	112.55 (102.55, 127.09)	107.61 (93.72, 125.30)	81.94 (64.20, 112.84)^∗^^†^	<0.001
EDA (pg/mL)	290.78 (240.30, 374.39)	366.19 (292.88, 426.27)^∗^	362.97 (307.47, 452.18)^∗^	0.002

Data are presented as means ± SD or medians (interquantile range (IQR)) for continuous variables and number (percentages) for categorical variables. SBP: systolic blood pressure; DBP: diastolic blood pressure; BMI: body mass index; WHR: waist-to-hip ratio; FPG: fasting plasma glucose; 2hPG: 2-hour postprandial plasma glucose; FIns: fasting plasma insulin; FC-P: fasting C-peptide; HOMA-IR: homeostasis model assessment-insulin resistance index; HbA1c: glycosylated hemoglobin c; TG: triglyceride; TC: total cholesterol; HDL-C: high-density lipoprotein cholesterol; LDL-C: low-density lipoprotein cholesterol; BUN: blood urea nitrogen; UACR: urinary albumin-to-creatinine ratio; eGFR: Estimated glomerular fltration rate.^a^Median (interquartile range); ^∗^*P* ≤ 0.05 vs. T2DM with normoalbuminuria; ^†^*P* ≤ 0.05 vs. group of T2DM with microalbuminuria.

**Table 3:** The significant correlations between EDA and clinical indices.

	All subjects (*n* = 248)		Control (*n* = 59)		All T2DM patients (*n* = 189)	
	*R* value	*P* value	*R* value	*P* value	*R* value	*P* value
Age	0.365^∗^	0.048	-0.091	0.491	0.181^∗^	0.012
BMI	0.200	0.289	-0.126	0.342	0.136	0.062
WHR	-0.279	0.136	0.089	0.501	-0.005	0.945
Duration of T2DM	—	—	—	—	0.044	0.552
SBP	-0.050	0.795	-0.113	0.395	0.085	0.245
DBP	-0.233	0.216	-0.068	0.611	-0.046	0.533
FPG	-0.298	0.110	0.097	0.465	-0.110	0.133
2hPG	-0.292	0.117	0.014	0.916	-0.093	0.204
FIns	0.409^∗^	0.025	-0.073	0.585	0.255^∗∗^	0.000
2hCP	0.060	0.752	0.050	0.707	0.084	0.248
HbA1c	-0.296	0.112	-0.010	0.941	-0.043	0.561
HOMA-IR	0.202	0.285	-0.048	0.716	0.219^∗∗^	0.002
HOMA-*β*	0.420^∗^	0.021	-0.126	0.341	0.203^∗∗^	0.005
TG	-0.498^∗∗^	0.005	0.066	0.618	0.002	0.977
HDL-C	-0.284	0.128	-0.061	0.646	0.026	0.719
ALT	0.124	0.516	-0.249	0.057	0.091	0.212
BUN	-0.123	0.516	0.097	0.464	0.062	0.399
Creatinine	-0.107	0.575	0.103	0.439	0.182^∗^	0.012
eGFR	0.130	0.495	0.073	0.585	-0.150^∗^	0.040
Log(UACR)	0.277^∗∗^	0.000	-0.176	0.181	0.222^∗∗^	0.002

**Table 4:** Stepwise multiple linear regression analysis with EDA as the dependent variable.

Independent variable	Regression coefficient (SE)	Standardized coefficient beta	*t*	*P*	95% CI
FIns	2.198 (0.548)	0.269	4.012	<0.001	1.119-3.278
HOMA-*β*	-0.035 (0.012)	-0.189	-2.906	0.004	(-0.058)-(-0.011)
Log (UACR)	35.521 (9.782)	0.223	3.631	<0.001	3.631-54.788

*CI:* confidence interval. The following independent variables were considered for the model: age, sex, smoking, DM duration, BMI, WHR, HbA1c, FPG, 2hPG, FIns, 2hIns, FC-P, 2hCP, TC, TG, LDL-C, HDL-C, HOMA-IR, HOMA-*β*, UA, BUN, creatinine, eGFR, and drug use. Only the variables that had a *P* < 0.05 were considered in the final fitted model.

**Table 5:** Results of multiple linear regression analysis for UACR in T2DM patients.

	Unadjusted			Adjusted by sex, smoking, hypertension, and drug use		
	*β*	*T*	*P*	*β*	*T*	*P*
Age	-0.050	-0.012	0.990	-0.309	-0.112	0.911
Duration of T2DM	0.050	0.171	0.864	-0.199	-0.621	0.535
BMI	-0.378	-0.042	0.967	-7.047	-0.770	0.442
WHR	-125.909	-0.285	0.776	-10.684	-0.024	0.981
SBP	5.547	2.630	0.009^∗^	4.707	2.157	0.032^∗^
DBP	-0.154	-0.056	0.955	-0.307	-0.111	0.912
FPG	46.533	3.162	0.002^∗^	46.621	3.177	0.002^∗^
2hPG	-7.352	-1.136	0.257	-9.272	-1.432	0.154
FIns	1.915	0.379	0.705	3.413	0.673	0.501
2hIns	1.776	1.025	0.307	1.001	0.567	0.571
FC-P	-25.991	-0.600	0.549	5.068	0.115	0.909
2hCP	-17.887	-1.124	0.262	-15.851	-0.975	0.331
HbA1c	-35.207	-1.961	0.051	-29.117	-1.595	0.112
HOMA-IR	-16.664	-1.087	0.278	-19.659	-1.274	0.204
HOMA-*β*	0.043	0.945	0.346	0.046	1.005	0.316
TG	-16.327	-0.539	0.590	-20.536	-0.677	0.499
TC	160.083	1.967	0.050^∗^	168.239	2.063	0.040^∗^
HDL-C	-60.244	-0.610	0.543	-110.276	-1.091	0.277
LDL-C	-146.934	-1.604	0.110	-161.613	-1.751	0.081
ALT	-1.373	-0.630	0.529	-0.522	-0.235	0.815
AST	2.830	0.711	0.478	1.984	0.488	0.626
UA	-0.212	-0.533	0.595	-0.237	-0.594	0.553
BUN	6.567	0.435	0.664	2.510	0.165	0.869
Creatinine	5.152	2.723	0.007^∗^	7.482	3.166	0.002^∗^
eGFR	-1.648	-0.914	0.362	0.236	0.109	0.913
EDA	0.543	2.475	0.014^∗^	0.495	2.248	0.026^∗^

**Table 6:** OR and 95% CI for albuminuria by the tertiles of ectodysplasin A in T2DM.

Models	EDA (pg/mL)	Individuals with and without albuminuria			
		*n*	OR	95% CI	*P*
Model 1	Lower tertile	63	1	Ref	Ref
	Middle tertile	63	2.219	1.075-4.582	0.031
	Upper tertile	63	3.739	1.788-7.820	<0.001

Model 2	Lower tertile	63	1	Ref	Ref
	Middle tertile	63	2.477	1.060-5.791	0.036
	Upper tertile	63	4.112	1.736-9.739	0.001

Model 3	Lower tertile	63	1	Ref	Ref
	Middle tertile	63	3.327	1.302-8.499	0.012
	Upper tertile	63	5.315	2.000-14.129	0.001

Abbreviation: CI: confidence interval; OR: odds ratio. Model 1: unadjusted. Model 2: adjusted for age, sex, duration, and blood pressure. Model 3: adjusted for age, sex, duration, blood pressure, BMI, WHR, smoking, hypertension, and drug use.

## Data Availability

The data will be available on request.

## Abbreviations

T2DM: Type 2 diabetes mellitu
DM: Diabetes mellitus
DKD: Diabetic kidney disease
EDA: Ectodysplasin A
SBP: Systolic blood pressure
DBP: Diastolic blood pressure
BMI: Body mass index
WHR: Waist-to-hip ratio
FPG: Fasting plasma glucose
2hPG: 2-hour postprandial plasma glucose
FIns: Fasting plasma insulin
FC-P: Fasting C-peptide
HOMA-IR: Homeostasis model assessment-insulin resistance index
HbA1c: Glycosylated hemoglobin c
TG: Triglyceride
TC: Total cholesterol
HDL-C: High-density lipoprotein cholesterol
LDL-C: Low-density lipoprotein cholesterol
BUN: Blood urea nitrogen
UACR: Urinary albumin-to-creatinine ratio
eGFR: Estimated glomerular filtration rate.
